# Editorial: The fruit fly, Drosophila, as a tool to unravel locomotor circuits

**DOI:** 10.3389/fncir.2023.1267789

**Published:** 2023-08-29

**Authors:** Wolfgang Stein

**Affiliations:** School of Biological Sciences, Illinois State University, Normal, IL, United States

**Keywords:** proprioception, larvae, fly, neuromuscular junction, mushroom body, neuromechanic, locomotor circuit

The neuronal control of movements has both fascinated and intrigued researchers for many decades. Body movement is a fundamental aspect of behaving organisms, is crucial for individual animal survival, and ensures the continuity of the species as a whole. As such, the neuronal control of body movement has been extensively studied across various species. Remarkably, despite the diverse range of animals and the multitude of movements they perform, the neuronal circuits responsible for controlling these movements are based on common functional principles and neuronal mechanisms (Pearson, 1993; Grillner and El Manira, 2020). These encompass the building blocks of network connectivity, intrinsic and synaptic neuronal properties, and, as recently demonstrated, molecular pathways as well (Meng and Heckscher, 2021).

An impressive Research Topic of research spanning various animal clades has fostered enduring collaborations and interactions among researchers exploring a wide range of species and behaviors. These include the undulating movements of *C. elegans* and lamprey, swimming in fish and sea slugs, walking in cats, crayfish, cockroaches, and stick insects, flying in locusts and flies, and even vocalization in amphibians, breathing in mammals, and chewing in crabs. These studies have also highlighted the unique characteristics of neuronal and circuit functioning that contribute to the generation of the diverse behavioral phenotypes.

Locomotion has consistently taken the lead in these studies due to the easily detectable and quantifiable behavioral output. While research on locomotor circuits in vertebrates has led to the identification and characterization of locomotion circuits in the spinal cord, invertebrate models have pioneered the roles of individual locomotor neurons and the mechanisms by which locomotor neurons and circuits function at a detailed level of resolution.

A more recent addition to studying locomotion is the fruit fly, *Drosophila melanogaster*. *Drosophila* offers insight into several under-researched aspects of locomotion that are challenging, if not unfeasible, to study in many other animals. These encompass the discovery of molecular and genomic pathways that enable locomotion and the comprehensive mapping of the underlying neuronal circuits, commonly referred to as the connectome. Much of our knowledge to date has been obtained by utilizing individual electrodes and single neuron recordings, but recent advances in identifying full circuit connectomes and creating genetic driver lines that target individual neurons have led to a remarkable expansion in our toolkit for studying the neuronal control of locomotion. These advancements have even provided a more in-depth exploration of pathologies and diseases that affect the locomotor system.

The surge of *Drosophila* research is fueled by this species' relatively short life span, compact genome, and a mostly established connectome. The availability of genetic tools for manipulating single neurons in combination with behavioral screens has further contributed to its popularity as a model for locomotion research. *Drosophila* shows two distinct life stages (larva and adult), with unique locomotion patterns. The objective of this topic is to shed light on recent research advancements that seek to unravel the development and dynamics of the neuronal circuits that underlie these patterns.

Two review articles provide insight into the larval stage and the neuronal control of its locomotion. Hunter et al. introduce those who do not usually use flies to the utility of the *Drosophila* larval locomotor network. The manuscript provides an overview of the locomotor circuit connectome and delves into a discussion about critical periods of development and interindividual variability in neural circuits, both of which are recent topics of interest in neuroscience.

In the second review, Kohsaka bridges neural control circuits and mechanical characteristics of the body. The manuscript provides an overview of the neuromechanics of the fly larva, detailing the mechanisms underlying locomotion. It also provides an entry point to a practical framework for scrutinizing mechanisms of locomotion in other animals and explores the latest advancements in soft robots that are inspired by larval locomotion.

The three original research articles in this Research Topic emphasize the benefits and usefulness of the *Drosophila* neuronal connectome, the capabilities offered by *Drosophila* genomics, and the usefulness of *Drosophila* in studying pathologies of neuronal locomotor circuits.

Greaney et al. use the larval connectome to map proprioceptor input and output synapses across several body segments, identifying neuronal features that distinguish proprioceptors and their synaptic connections from somatosensory neurons. A comprehensive map of how proprioceptor projections are organized centrally is provided, opening new avenues to study downstream proprioceptive processing circuits and to explore developmental mechanisms that drive proprioceptor connectivity.

Eidhof et al. explore the interactions between ataxia-associated DNA repair genes and precise motor control, using the mushroom bodies of the central brain of adult flies. The manuscript highlights the contributions of mushroom body DNA damage to deficits of startle-induced and spontaneous motor behaviors after introducing a loss-of-function in DNA repair genes. The study also suggests that aberrant glutamate signaling may play a role in conferring motor control circuit vulnerability in DNA repair disorders, including autosomal recessive cerebellar ataxia.

The final original research article by Kinold et al. examines the molecular underpinnings of axonal innervation of the neuromuscular junction during metamorphosis. This developmental period involves extensive remodeling of the neuromuscular system to enable a new set of behaviors as the fly undergoes its transformation from larva to adult. The study underscores the significance of re-expressing a transmembrane protein that attracts motor axons, which helps prevent misinnervation and subsequent pathologies of the muscles following metamorphosis.

In conclusion, the studies showcased in this Research Topic shed light on aspects of the intricate neuronal control of locomotion in *Drosophila* from a molecular and circuit mechanism point-of-view. The use of *Drosophila* offers new perspectives and opens up new avenues for studying the neural control of movements in health and disease. Studies in *Drosophila* will complement existing studies, help unravel the complexities of locomotion, and facilitate an in-depth understanding of motor system organization and function.

## Author contributions

WS: Conceptualization, Funding acquisition, Supervision, Validation, Visualization, Writing—original draft, Writing—review and editing.
